# HPV-positive cancers at uncommon sites: a narrative review

**DOI:** 10.3389/or.2026.1737332

**Published:** 2026-03-11

**Authors:** Lorenzo Agoni, Orges Spahiu, Dukagjin M. Blakaj

**Affiliations:** 1 Unit of Obstetrics and Gynecology, Fondazione Poliambulanza Istituto Ospedaliero, Brescia, Italy; 2 Radiation Therapy Unit, Mother Teresa University Hospital Center, Tirana, Albania; 3 Department of Radiation Oncology, The Ohio State University Comprehensive Cancer Center, Columbus, OH, United States

**Keywords:** cancer, genotypes, HPV, papillomavirus (HPV), uncommon sites

## Abstract

Human papillomavirus (HPV) usually infects the anogenital and the oropharyngeal areas. HPV may lead to cancer at these sites, most notably at the uterine cervix. Less frequently, vaginal, vulvar, anal and penile cancers may also arise. HPV may be also found, sometimes, in other cancers, such as lung, breast, bladder, esophageal cancers and others, which are not typical sites for HPV-related cancers. HPV spreads easily throughout the body and it has an intrinsic carcinogenic potential, which it may operate on organs which are distant from the genital area, although with a limited incidence of disease. It has been debated whether such occurrence has a casual or causative significance. In this systematic review we summarize the evidence and the pitfalls of these uncommon HPV-positive cancers, with particular emphasis on HPV genotypes distribution.

## 1 Introduction

Human papillomavirus (HPV) infection, which is the most common sexually transmitted infection worldwide, usually targets organs at the anogenital area and organs of the head and neck area, which are those involved in sexual intercourse and where mucous epithelia, which are the target of the virus, are found. The actual incidence of the infection is unknown, but it is estimated that approximately 80%–90% of people get infected by HPV at least once within the 45 years of age (1). The prevalence of the infection accounts to an average of 11,7% worldwide but may vary according to country and age groups, ranging from approximately 1% in some regions of Asia to over 30% in some regions of Africa (2).

HPV is a well-known carcinogenic agent and it’s the major determinant of cervical cancer. Likely, 98%–100% of cervical cancers depend on HPV infection. According to the “Human Papillomavirus and related diseases Report” (2) issued by IARC in 2023, approximately 600,000 new cases of cervical cancer are diagnosed every year globally. Also, HPV may cause cancer in other organs of the anogenital and head and neck areas. While we can count 280,000 oral cavity cancers, 180,000 laryngeal cancers, 140,000 oropharyngeal cancers, 44,000 vulvar cancers, 34,000 penile cancers, 29,000 anal cancers, 18,000 vaginal cancers new diagnoses yearly worldwide, HPV may have a role in a fraction of these malignancies. Specifically, HPV may be the cause of the disease in 42,000 (30%) of the oropharyngeal cancers, 29,000 (100%) of the anal cancers, 18,000 (53%) of the penile cancers, 14,000 (78%) of the vaginal cancers, 11,000 (25%) of the vulvar cancers, 5,900 (2%) of the oral cavity cancers, 4,100 (2%) of the laryngeal cancers (3). These are well-known HPV-related cancers. Interestingly, HPV may be found also in a small percentage of other cancers for which the relationship with HPV has not been clearly elucidated yet.

With this systematic review we focus on these less common HPV-positive cancers, collect the available evidence and try to put it into perspective.

## 2 HPV carcinogenicity

Among the over 200 genotypes of HPV identified so far, IARC classified twelve of them as carcinogenic (Group 1): HPV-16, HPV-18, HPV-31, HPV-33, HPV-35, HPV-39, HPV-45, HPV-51, HPV-52, HPV-56, HPV-58, and HPV-59; HPV-68 is classified as probably carcinogenic (Group 2A) and a few others, specifically HPV-26, HPV-30, HPV-34, HPV-53, HPV-66, HPV-67, HPV-69, HPV-70, HPV-73 and HPV-82, are classified as possible carcinogenic (Group 2B) (4), mainly because they belong to the same phylogenetic families as the carcinogenic genotypes. The other HPV genotypes are generally considered not carcinogenetic, although a few genotypes, particularly HPV-6 and HPV-11, can cause condylomas, which is a benign condition characterized by verrucous outgrows on the genitalia.

The mechanism through which HPV exerts its carcinogenetic potential is well known although not fully characterized. Briefly, upon infection, the viral proteins interact with the host cell proteins to ensure the replication of the virus. Two specific viral proteins are important to the carcinogenetic process: E6 and E7. They interfere with two cellular proteins, the latter interact with Rb protein and elicits uncontrolled proliferation, while the former inhibits the actions of the oncosuppressor p53. Both proteins are tightly regulated by another viral protein, E2. The gene for this protein often gets disrupted during the process of integration, which leaves E6 and E7 completely free to be overexpressed, eliciting cellular proliferation and immortalization. Integration is not a required step in viral replication. In fact, disruption of the viral genome during integration interrupts the vital cycle of the virus. Thus, integration is to be considered a biological mistake in terms of viral survival. When integration occurs, the carcinogenetic process begins and it starts with the formation of a pre-neoplastic lesion, also called dysplasia.

An interesting step of HPV carcinogenicity involves the p16 protein, which is a cyclin-dependent kinase inhibitor that normally blocks CDK4/6, preventing phosphorylation of Rb. Hypo-phosphorylated Rb binds and sequesters E2F transcription factors, blocking G1 to S cell-cycle progression. In HPV driven cells p16 is expressed as a compensatory response to unchecked E2F activity. When HPV activity becomes unopposed, such as in dysplasia, p16 is overexpressed (5). Thus, p16 has been used as a biomarker for dysplasia progression. For this reason, in recent years, p16 has been evaluated for being introduced into the algorithm for cervical cancer screening (6, 7).

HPV infects the basal layer of mucosal epithelia and here the abovementioned process starts. At the beginning it may involve only the deep layers of the mucosa and form the so called LSIL; by time, the process can extend to the superficial layers and form the so called HSIL. Then, when the lamina propria at the basal layer gets disrupted by the action of these proliferating cells, we can define the lesion as invasive carcinoma. This process is similar regardless of the organ where it occurs, given that there is a mucosal epithelium. It is still debated why in some organs, such as cervix and anus, it seems to be more efficient than in other organs.

## 3 Methods

On date 21st August 2025, we searched the PubMed database for articles mentioning HPV and specific cancers in the title or abstract using the search string: (HPV[Title/Abstract]) AND ((* cancer[Title/Abstract]) OR (* carcinoma[Title/Abstract])), where the star was replaced with a specific organ from the 39 cancers listed in Table 1. We did not constrain any timeframe for the search, thus including all available evidence. We focused on the PubMed database.

**TABLE 1:** Number of publications retrived by scientific literature search.

Type of cancer^(a)^	PubMed search
1. Anal	1173
2. Bladder	148
3. Bone	2
4. Breast	498
5. Brain	7
6. Cervical	20620
7. Colon	70
8. Endometrial	148
9. Esophageal	201
10. Eye/ocular/eyelid	6
11. Gastric	72
12. Head and neck	2598
• Ear	3
• Hypopharyngeal	60
• Laryngeal	253
• Nasopharyngeal	164
• Oral cavity	121
• Oropharyngeal	2768
• Parathyroid	1
• Salivary gland	14
• Sinonasal	93
• Tongue	113
• Tonsil	74
13. Hepatobiliary	0
14. Hepatocellular	89
15. Kidney	11
16. Lung	367
17. Ovarian	202
18. Pancreatic	16
19. Penile	504
20. Prostate	254
21. Rectal	31
22. Skin	446
23. Testicular	16
24. Thyroid	35
25. Vaginal	193
26. Vulvar	443
Total	31814

a Different types of cancer, mentioned in the text, are listed in column 1. In column 2 the number of scientific articles retrived by the literature search are shown for each cancer type (see Method section).

The typical sites of HPV-related cancer (anal, cervical, head and neck, oropharyngeal, penile, rectal, skin, vaginal and vulvar cancers) were then excluded from further analysis. On the remaining 30 sites a first screening was performed to exclude articles focused on preclinical data, benign disease, unknown primary site disease, metastatic disease, antibody serum levels, blood circulating HPV, detection methods, vaccines, letters and comments, guidelines, awareness studies, quality of life studies, hypothesis articles, articles lacking the prevalence of HPV in the sample, duplicated articles and retracted articles. A second screening was performed to separate research papers from reviews, including systematic reviews and metanalysis. This was performed because research papers show the primary evidence while reviews collect the already published evidence from the research papers, sometimes performing further analysis on the data, for example in the systematic reviews. Thus, we differentiated the two levels of investigation. The results are presented in Table 2.

**TABLE 2:** Number of scientific publications categorized by publication type.

Type of cancer^(a)^	PubMed search	1st screening	2nd screening	
			Research	Reviews
1. Bladder	148	72	55	17
2. Bone	2	1	0	1
3. Breast	498	152	124	28
4. Brain	7	0	0	0
5. Colon	70	17	13	4
6. Endometrial	148	24	21	3
7. Esophageal	201	104	80	24
8. Eye/ocular/eyelid	6	6	6	0
9. Gastric	72	21	11	10
10. Head and neck	​	​	​	​
• Ear	3	3	2	1
• Hypopharyngeal	60	16	15	1
• Laryngeal	253	80	67	13
• Nasopharyngeal	164	33	30	3
• Oral cavity	121	22	17	5
• Parathyroid	1	0	0	0
• Salivary gland	14	4	4	0
• Sinonasal	93	50	44	6
• Tongue	113	34	30	4
• Tonsil	74	21	19	2
11. Hepatobiliary	0	0	0	0
12. Hepatocellular	89	1	1	0
13. Kidney	11	2	0	2
14. Lung	367	117	95	22
15. Ovarian	202	33	26	7
16. Pancreatic	16	0	0	0
17. Prostate	254	61	46	15
18. Testicular	16	7	2	5
19. Thyroid	35	7	7	0
**Total**	**3038**	**888**	**715**	**173**

Total articles per column are in bold.

a Different types of cancer, mentioned in the text, are listed in column 1. Number of included articles are listed in column 2. In column 3 and 4 the number of articled categorized by publication type are listed.

Potentially eligible articles were retrieved independently by two investigators (LA and OS), and any discrepancies were resolved by a third investigator (DMB).

From the resulted articles we retrieved the available data on HPV prevalence for each specific cancer type and, when available, also the prevalence of specific HPV genotypes. Then, we presented the data for each cancer site starting by the best available evidence, such as systematic reviews and metanalysis. When systematic reviews are available, we present the results of the most recent ones. When no systematic reviews are found for a specific cancer site, we present the most relevant available articles, including case reports and clinical research papers, although we are aware of the overall lower strength of the evidence for such publications.

## 4 Results

**FIGURE 1:**
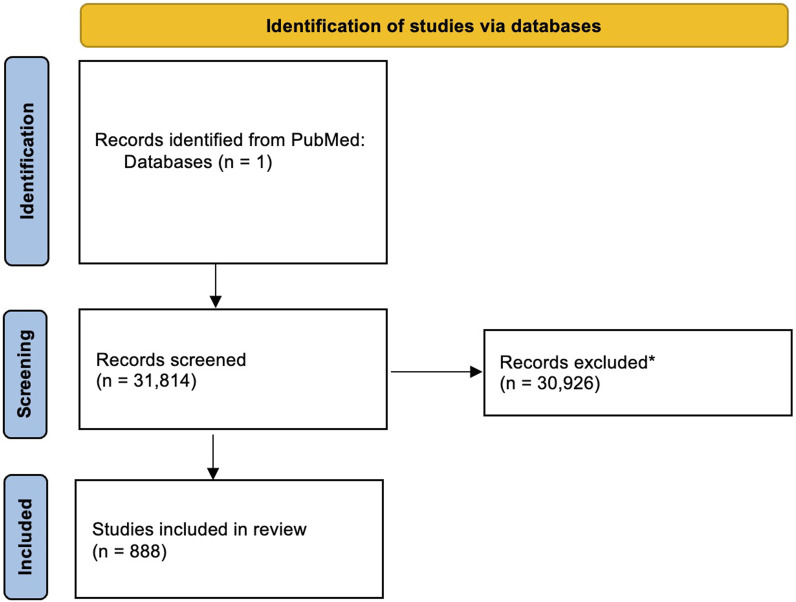
The PRISMA flowchart showing search methodology and results. *exclusion criteria: Preclinical; Benign disease; Metastatic disease; Retracted articles; Unrelated to specific topic; Circulating HPV; Serum levels of antibodies; Unknown primary site; Letters and comments; Well known HPV-related cancers and cutaneous HPVs (highlighted in red); Vaccine and immunotherapy strategies; Guidelines; Awareness studies; QoL studies; Duplicated papers; Methods of HPV detection; Hypothesis papers. Source: Page MJ, et al. BMJ 2021; 372:n71. doi: 10.1136/bmj.n71. This work is licensed under CC BY 4.0. To view a copy of this license, visit https://creativecommons.org/licenses/by/4.0/.

The database search for articles regarding HPV and specific cancer sites of 39 different locations resulted in 31,814 papers. A first screening, and the exclusion of common HPV-related cancer sites, allowed to select 888 papers for further analysis, distributed on 30 cancer sites. Results of the search are shown in the PRISMA 2020 flowchart diagram in Figure 1. Cancer sites with the highest number of articles were breast, lung and esophageal cancers, with 152, 117 and 104 papers, respectively. Cancer sites which retrieved no results were brain, parathyroid, hepatobiliary and pancreatic cancers.

We now review each cancer site in alphabetical order, presenting the best available evidence to confirm or confute an HPV carcinogenic role.

### 4.1 Bladder cancer

Bladder cancer is the 9th cancer for incidence worldwide accounting for over 600,000 new cases in 2022, and a 5.6/100,000 incidence (ASR), according to GLOBOCAN (8).

Bladder cancer is more common in high-income countries, such as Europe and North America. There is a higher incidence in men than in women. Risk factors such as diet, smoking and environmental and occupational exposure to chemical substances are well-known risk factors for developing the disease.

Histology of bladder cancer can be differentiated in urothelial, which is the predominant histological type accounting for ∼90%, is mainly related to chemical exposure, whereas squamous cell carcinoma (5%) is associated with chronic inflammation and persistent infections (such as Schistosoma spp. in Africa). Adenocarcinoma (2%), sarcoma, and small cell carcinoma are fewer incident forms.

Our database search for bladder cancer and HPV retrieved 72 articles, among which 13 reviews and 4 systematic reviews with metanalysis. The most advanced analysis is first authored by Muresu and published in 2022 (9). Their analysis included 46 studies on bladder cancer, including 3975 patients, with various bladder cancer histology such as urothelial carcinoma, squamous cell carcinoma, transitional cell carcinoma and adenocarcinoma. In this study the prevalence of HPV in bladder cancer was 19%, in line with previous metanalyses, ranging from 0% to 83% across the studies. A higher prevalence of 36.5% for HPV has been shown in squamous cell carcinoma.

The most prevalent genotype was HPV-16 (45.1%), followed by HPV-18 (31.9%), HPV-6 (5.2%), HPV-11 (3.5%) and other HR-HPV genotypes (14.2%).

This study showed a significant association between HPV and bladder cancer with an odds ratio (OR) of 7.84, confirming the findings of previous meta-analyses on the topic.

This metanalysis includes a few studies that evaluate p16 as a biomarker. A 2010 study by Gould (10) considered 18 inverted papillomas of the urinary bladder among which 11 resulted positive for p16. However, only 2 cases were HPV positive. A 2012 study by Alexander (11) considered 42 cases of squamous cell carcinoma of the urinary bladder and 27 cases of urothelial carcinoma with squamous differentiation. HPV was not detected in any of the 42 cases while p16 expression was detected in 13 cases (31%) of squamous cell carcinoma and 9 cases (33%) of urothelial carcinoma with squamous differentiation. A subsequent study by the same Author (12) considered 36 cases of bladder adenocarcinomas. Among these, 24 (67%) cases were positive for p16 staining but no HPV was detected. A 2014 study by Kim (13) included 35 patients with bladder urothelial cancer with squamous differentiation and compared with 12 patients with squamous metaplasia of the bladder. HPV detection rates were approximately 2-fold higher in the study than the control group (17.1% versus 8.3%, respectively), but the difference was not statistically significant. Overexpression of p16 was detected in 16 (45.7%) cases in the study group and 1 (8.3%) case in the control group (p = 0.034). The Authors conclude that there is no correlation between p16 expression and HPV in these cancers and that p16 cannot be used as a surrogate marker for HPV infection in bladder cancer. Interestingly, a study by Steinestel (14) correlates the p16 overexpression in these tumors, particularly urothelial carcinoma *in situ*, with epithelial-to-mesenchymal transition (EMT) but not with HPV.

### 4.2 Bone cancer

Primary Bone cancer is a rare as it accounts for 0.2% of all cancers. It is more common in children totaling 5% of children’s malignancies (15–17). The bone malignancies are encountered often in the form of metastatic diseases from distant primary sites, such as breast, prostate, lung or cervix etc. and are more frequent, accounting for 5%–7% of cancer patients (18).

Here we focus on the primary bone carcinoma, which is an extremely rare occurrence and in 1%–2% of cases involves skull bones (17). Our search retrieved only one review mainly focused on middle ear cancer and temporal bone carcinoma. High-risk HPV-related squamous cell carcinoma in the temporal bone is a rare but noteworthy tumor subtype (19). This study included 131 cases of temporal bone squamous carcinoma. Among these 18 (13.7%) cases overexpressed p16, 5 of which were also HPV positive. The positive predictive value (PPV) of p16 positivity for high-risk HPV infection was 27.8%. Thus, immunohistochemistry for Rb was used to increase PPV for HPV detection. The combination of p16/Rb partial loss pattern showed excellent reliability with a PPV of 100%.

A single study shows an HPV prevalence of 21% in temporal bone squamous carcinoma (20).

The actual origin of the temporal bone squamous cell carcinoma is the squamous epithelium that cover the external auditory canal. The relationship between middle ear carcinoma and HPV will be explored in a specific paragraph of this article.

### 4.3 Breast cancer

Breast cancer is the 2nd cancer for incidence worldwide accounting for over 2,000,000 new cases in 2022, and a 46.8/100,000 incidence (ASR), according to GLOBOCAN (8). It constitutes the most common malignancy in women.

Breast cancer is more common in high-income countries, such as Europe, North America and Australia. On the other hand, mortality is higher in low-income countries, such as in Africa and South America.

A few risk factors are known for the disease: genetic predisposition (such as BRCA gene mutations), age, obesity, alcohol consumption and smoking, family history, early menarche and late first pregnancy, and postmenopausal hormone therapy, radiation exposure. However, a significant fraction of breast cancers occurs in women with no identifiable risk factors. For this reason, scientists have been looking for different explanations for carcinogenesis in breast cancer. HPV is one of those agents that have been more often implied in this effort, although conflicting results have been reported in the literature.

Our database search retrieved 152 articles among which 28 reviews.

A 2024 comprehensive review by Rossi (21) helps in shedding light on this controversial topic. They identified 68 studies for systematic review, totalizing 5907 patients. In their study set, the HPV prevalence was 18%, but it ranged from 0% to 84% across the studies. HPV-16 was the most detected HPV genotype in breast cancer, followed by HPV-18 and HPV-33. Apparently, no difference in tumor characteristics or prognosis was found when compared HPV-positive to HPV-negative cases. However, pooling cases (HPV-positive breast cancer) and controls (HPV-positive benign disease) together, there was an HPV prevalence of 24% in the cases and of 8% controls with a significant p-value (p < 0.0001). This paper does not explore the role of p16 as a biomarker for HPV infection or progression in breast dysplasia or cancer. However, p16 has been studied as a marker for tumor progression and response in breast cancer (22–24), even though it is likely unrelated to HPV presence (25).

The studies included in this systematic review consider multiple histological types being ductal carcinoma/carcinoma of no special type the most commonly reported. Some studies are focused on specific histological types, such as triple negative breast cancer in which HPV-positivity was generally higher than non-triple negative cancers (26–28). One case reported positivity to HPV-18 and HPV-33 in a lymphoepithelioma-like carcinoma of the breast (29).

### 4.4 Colon cancer

Colon cancer is the 4th cancer for incidence worldwide accounting for over 1,000,000 new cases in 2022, and a 10.7/100,000 incidence (ASR), according to GLOBOCAN (8). It is more common in high-income countries, such as Europe, North America, Russia, and Australia.

Typical risk factors for colon cancer include age over 50, a personal or family history of the disease, inflammatory bowel conditions, and certain lifestyle choices such as a diet high in red and processed meats, physical inactivity, obesity, smoking, and alcohol consumption.

Our database search retrieved 19 articles among which 4 reviews.

The only metanalysis within the results was first authored by Damin (30) and was published in 2013. In this study 16 articles were included, comprising 1436 patients with colon adenocarcinoma. The prevalence of HPV in colon cancer ranged from 0% to 84%, with a pooled value of 31.9%.

Overall, there was a significant increased risk of 4.054 (95% CI: 1.79–9.14) for HPV infection in the tumor samples compared with the nontumor controls.

HPV-18 was the most prevalent genotype (73%) in the studies from Asia, while HPV-16 was the most prevalent genotype (58%) in the studies from other continents. Altogether, HPV-18 was found in 52.85% of cases, HPV-16 in 37.85%, HPV-33 in 17.07%. Since this metanalysis was published, only 3 additional more recent articles to date were found by our search. All 3 of them were performed in Iran on 100 (31), 130 (32) and 80 (33) colon adenocarcinoma patients, respectively. None of these 3 studies was able to show any correlation between HPV and colon cancer, because two of them could not detect any HPV neither in cases nor in controls, while the third found HPV to a low prevalence (6.25%).

The role of p16 was not explored in the mentioned studies. No studies were found to correlate p16 expression to HPV positivity in colon cancer. The overexpression of p16 has been rarely investigated in colon cancer (33), while its methylation status appears to attract more interest, at least in in vivo studies (34–36).

### 4.5 Endometrial cancer

Corpus uteri cancer is the 15th cancer, and the 6th in women, for incidence worldwide accounting for over 400,000 new cases in 2022, and an 8.4/100,000 incidence (ASR), according to GLOBOCAN (8). It is more common in high-income countries, such as parts of Europe, North America, Russia, and Australia.

Typical risk factors for endometrial cancer include age, with increased risk in women over 50, and hormonal factors such as prolonged unopposed estrogen exposure. Obesity is a significant risk factor due to higher estrogen levels produced by adipose tissue. Other factors include a history of irregular menstrual cycles, late menopause, and genetic conditions like Lynch syndrome.

Histologically, the endometrial carcinoma is usually an endometroid adenocarcinoma. A smaller fraction of endometrial cancer shows serous or clear cell differentiation or even a squamous differentiation.

Our database search retrieved 24 articles among which 3 reviews. A systematic review and metanalysis was published in 2014 and first authored by Olesen (37). Twenty-nine studies were included in their analysis for a total of 1026 patients with endometrial cancer. The pooled prevalence of HPV DNA was 10.0% (95% CI: 5.2–16.2), with a wide range, from 0% to 61%. Although the odds ratio was 1.43 the confidence intervals (95% CI: 0.68–3.00) showed that this increased risk was not statistically significant. It is interesting that HPV prevalence for squamous differentiation (256 cases) was generally higher, 12.4% on average when compared to endometrioid adenocarcinoma for which the HPV prevalence was 8.3%. The serous histological subtype comprised only 21 cases and was not considered for further analysis. The PCR probes used for finding HPV were very heterogenous across studies, ranging from HPV-16 and HPV-18 to a full set of more than 20 genotypes. Thus, information about HPV genotypes distribution is fragmentary and possibly meaningless. In conclusion, there was no evidence of a correlation between HPV and endometrial cancer and the presence of the virus within the cancer tissue is considered only as a passenger effect.

Since then, 5 more studies were published. Among these, the study by Wu (38) is particularly interesting as it is a cohort population study, from Taiwan, comprising 472,420 female patients with HPV infection and 944,840 without HPV infection. The hazard ratio for developing endometrial cancer in HPV positive patients was 1.588 (95% CI: 1.335–1.888) and statistically significant. The HPV genotypes were not explored in this study. As far as histology is concerned, 0.86% of the HPV-positive cases developed an endometriod adenocarcinoma as compared to 0.50% of the HPV-negative cases (HR 1.671; 95% CI: 1.376–2.029) and a serous adenocarcinoma the 0.08% of the HPV-positive cases as compared to 0.06% of the HPV-negative cases (HR 1.450; 95% CI: 0.791–2.656). Thus, HR results statistically significant only in the endometrioid adenocarcinoma cases.

Overexpression of p16 is not evaluated in any of the mentioned studies. In fact, p16 overexpression in endometrial cancer has been considered independent from HPV (39) and even useful to differentiate endometrioid endometrial cancer from endocervical cancer and serous endometrial cancer because the former usually presents a patchy positivity while the two latter show a strong diffuse positivity (40).

### 4.6 Esophageal cancer

Esophageal cancer is the 11th cancer for incidence worldwide accounting for over 500,000 new cases in 2022, and a 5.0/100,000 incidence (ASR), according to GLOBOCAN (8). It is more common in Asia, Russia and sub-Saharan Africa.

Typical risk factors for esophageal cancer include smoking and alcohol consumption, gastroesophageal reflux disease and Barrett’s esophagus. Additionally, obesity, poor diet and a history of achalasia or previous radiation therapy can contribute to higher risk.

Our database search retrieved 104 articles among which 24 reviews. The most recent comprehensive review was published by Bhatt in (41). In this review the last few metanalysis on the topic are reported and commented. A metanalysis of 21 case–control studies, published by Liyanage in (42), found that esophageal squamous cell cancers were three times more likely to have HPV DNA than controls (pooled OR 3.04, 95% CI: 2.20–4.20). Another 2013 metanalysis, on 68 studies involving 5755 esophageal squamous cell cancer patients, on high-risk subtypes 16 and 18 specifically showed increased risk of esophageal cancer only in HPV-16 infection (OR 3.55, 95% CI: 2.05–6.14) while this was not apparent for HPV-18 (43). This was consistent with a 2014 metanalysis, on 132 studies accounting for 12037 esophageal squamous cell cancer patients, that showed an increased risk for HPV infection, especially HPV-16 (OR 2.69, 95% CI: 2.05–3.54 and OR 2.35, 95% CI: 1.73–3.19) (44). The average prevalence of HPV was 24.8%, ranging from 2.8%, in France, to 59.4%, in Mexico. A 2021 meta-analysis, published by Petrelli, found HPV prevalence in esophageal squamous cell cancer cases at 18.2% (95% CI: 15.2%–21.6%) with a pooled OR of 3.81 (95% CI: 2.84–5.11) (45). These analyses considered only HPV-16 and HPV-18. Thus, any genotype consideration cannot be fully explored. A few studies detected also other genotypes as well. For example, in a 2024 study by Ndemela (46), 63/118 (53.3%) esophageal squamous cell cancer samples tested positive for high-risk HPV. Among these, HPV-16 was the most prevalent 41/63 (65.1%), followed by HPV-18 (HPV18) 15/63 (23.8%), and the rest 7/63 (11.1%) were other hrHPV genotypes. This was confirmed in a study by Zhang (47) in which the HPV genotypes were identified in esophageal carcinoma (without specify the histological subtype) were, in decreasing order of prevalence: HPV-16, HPV-58, HPV-18, HPV-33, HPV-31 and HPV-11. In another study by Wang (48) HPV-16 and HPV-57 were the most common types, followed by HPV-26 and HPV-18. This study included 34 esophageal adenocarcinomas and 401 esophageal squamous cell carcinomas. HPV was detected in 52.9% of esophageal adenocarcinomas and 66.7% of esophageal squamous cell carcinomas (p = 0.200). Interestingly, one study (49) found lrHPV (low-risk Human Papilloma Virus) more common than hrHPV (high-risk Human Papilloma Virus) in esophageal squamous cell cancer, with a global HPV prevalence of 50%.

The metanalysis published by Bhatt (41) addresses also the role of p16 in esophageal cancer. It is highlighted that a few dedicated studies found little or no correlation between p16 overexpression and HPV. For example, a systematic metanalysis by Michaelsen (50) published in 2014 found no difference in p16 expression between HPV-positive and HPV-negative esophageal cancer samples.

### 4.7 Eye cancer

This definition comprises the search keywords “eye,” “ocular,” “eyelid” cancers but not “melanoma.” Eye cancer is a rare disease with a global incidence of 0.49/100,000 new cases per year (51). It is more common in Sub-Saharan Africa when compared to other continents with an incidence of 4.06/100,000. The most common histological type of eye cancer is melanoma, which is not considered in this review.

Known risk factors for eye cancer are UV light exposure, smoking and alcohol consumption.

Our database search retrieved 6 articles and no reviews.

In 2003 Montoya-Fuentes (52) analyzed 51 samples from ocular tissues of retinoblastoma patients and of 6 controls enucleated for non-neoplastic reasons. Forty-two (82.3%) of the 51 samples were HPV-positive. Genotyping was performed and the results are that HPV-6 was found in 40 cases (95.2%), HPV-33 in 16 (38.1%), HPV-11 in 4 (9.5%) and HPV-31, 35 and 51 each in one case (2.3%). All controls were negative for HPV-DNA.

A 2015 article by Galor (53) considered 27 cases of ocular surface squamous neoplasia and found 21 cases positive for HPV. The HPV genotypes identified included HPV-16 in 10 tumors (48%), HPV-31 in 5 tumors, HPV-33 in 1 tumor, HPV-35 in 2 tumors, HPV-51 in 2 tumors, and unidentified HPV in 3 tumors.

A 2013 study from Mozambique (54) analyzed 22 consecutive biopsies of intraepithelial neoplasia (2 low-grade and 9 high-grade cases), squamous cell carcinoma of the conjunctiva (8 cases), and benign conditions (3 cases). In 11 (57.9%) cases of intraepithelial neoplasia or carcinoma HPV-DNA was found, while benign biopsies were negative for HPV. Then, specific search for HPV-16 or HPV-18 resulted in positivity for HPV-16 in two cases and HPV-18 in one case, while other HPV genotypes were not tested.

A 2023 study by Yang (55) on a Korean population considered 34 eyelid sebaceous gland carcinomas and 12 eyelid squamous cell carcinomas. HPV was detected in 4 (11.7%) sebaceous gland carcinomas and 6 (50%) squamous cell carcinomas. The HPV genotypes identified in sebaceous gland carcinomas were HPV-16 in 2 cases, HPV-18 in one case and HPV-56 in one case. The HPV genotypes identified in squamous cell carcinomas were HPV-16 in 4 cases, HPV-51 and HPV-68 in one case each.

In 2023 Kakkar (56) showed two cases of eyelid squamous carcinoma which was positive for HPV-16.

In 2024 Sanchez (57) reported a case of HPV positive eyelid squamous carcinoma in a patient with history of breast cancer. The cancer tissue was positive for both HPV-16 and HPV-18.

Few of the mentioned papers evaluated p16 status. For example, in the study by Yang (55) among 30 sebaceous gland carcinomas evaluated for p16, 19 (63.3%) showed positive, and 11 showed negative staining, while among 9 squamous cell carcinomas evaluated for p16, only 3 (33.3%) showed positive staining for p16. Among samples showing positive staining in p16, HPV-positive rates were 0.0% (0/19) in sebaceous gland carcinomas and 100% (3/3) in squamous cell carcinomas.

The study by Kakkar (56) showed p16 positivity in two cases of eyelid squamous cell carcinomas.

### 4.8 Gastric cancer

Gastric cancer is the 5^th^ cancer for incidence worldwide accounting for almost 1,000,000 new cases in 2022, and a 9.2/100,000 incidence (ASR), according to GLOBOCAN (8). It is more common in Asia, Russia and South America.

Typical risk factors for gastric cancer include chronic *Helicobacter pylori* infection, which causes long-term inflammation of the stomach lining, dietary factors such as high salt intake, smoked and poorly preserved foods.

Our database search retrieved 21 articles among which 10 reviews.

Zeng (58) reported that in 2016, a total of 15 case-control studies, including 12 studies on Chinese patients, and a metanalysis showed that HPV infection increased the risk of gastric cancer by 7.39 times [95% confidence interval (CI) of summary odds ratio (sOR) = 3.88–14.1]. A systematic review and metanalysis was published by Wang in 2020 (59). The study was focused on non-EBV infections of the stomach, including CMV, hepatitis viruses and HPV. A total of 41 studies were included in the analysis. Fourteen of these studies were focused on HPV, accounting for 901 patients. Association between HPV infection and the risk of gastric cancer was observed (OR = 1.53, 95% CI: 1.00–2.33, random model, heterogeneity P < 0.002). The studies in which genotyping was performed identified statistically significant differences between cancer cases and controls for HPV-16, HPV-18, HPV-33, and HPV-39.

Since 2020, only two more articles were found on the topic. One article published by Wu (60) in 2023 is focused on HPV-16 integration in gastric cancer. Out of 361 gastric cancer samples, 10 were positive for HPV. HPV-16 was found in 5 out of 10 of such samples. The remaining 5 HPV-positive samples were not genotyped.

In 2025 Mazurek (61) published an article on EBV and HPV on gastric cancer without finding any significant impact of either infection in 37 patients, as only 4 HPV-positive cases were identified. The histological subtypes are not mentioned in these metanalyses. Moreover, non of the mentioned papers explored p16 overexpression in their analyses. However, Ding (62) in 2010 reported high expression (59%, 10/17) of p16 in gastric cardia cancers while only a limited HPV-16 positivity (29%, 5/17).

### 4.9 Head and neck cancers

#### 4.9.1 Ear cancer

Ear cancer is a rare malignancy with an approximative incidence of 0.1/100,000 cases per year worldwide (63).

Because is a rare disease, risk factors are not well characterized. However, they may include chronic ear infections, sun exposure, smoking, radiation, and exposure to industrial chemicals.

Our database search retrieved 3 articles among which 1 review.

In this 2016 review by Marioni (64), two research articles were cited, both from 1997, one by Jin (65), the other by Tsai (66). Both studies investigated the influence of HPV-16 and HPV-18 on middle ear squamous carcinoma. In the latter study 14 samples were analyzed, and the result highlighted the presence of both HPV-16 and HPV-18 in 5 cases and HPV-16 alone in 6 cases. The former study reported finding that 8 out of 9 middle ear cancers were

HPV-positive; HPV-16 was detected in four squamous cancers and the one adenocarcinoma included in the series, while HPV-16 and HPV-18 coexisted in 3 squamous carcinomas. Further genotyping was not performed.

Since then, no other article has been published on the topic.

None of the mentioned papers explore p16 overexpression as a biomarker.

#### 4.9.2 Hypopharyngeal cancer

Hypopharyngeal cancer is the 25th cancer for incidence worldwide accounting for approximately 86,000 new cases in 2022, and a 0.89/100,000 incidence (ASR), according to GLOBOCAN (8). It has a varied distribution globally, being more common in parts of Europe, India, Russia, Africa and South America.

Typical risk factors for hypopharyngeal cancer include smoking, alcohol consumption, and exposure to certain industrial chemicals, chronic irritation from poor oral hygiene or repeated infections may also contribute to risk.

Our database search retrieved 16 articles among which 1 review.

A metanalysis was published by Shi in 2022 (67), accounting for 18 studies and 6098 hypopharyngeal squamous cell cancer patients. They found an HPV prevalence ranging from 6.4% to 82%. However, no genotyping was mentioned. The main result of their study was that there was a statistically significant difference in overall survival between the HPV DNA-positive group when compared to HPV DNA-negative group (HR = 0.61, 95% CI [0.54, 0.69], p = 0.0001), and HPV infection was beneficial to the survival of hypopharyngeal cancer patients. This result is confirmed by a 2022 clinical trial by Yang (68) who studied 531 patients receiving radiotherapy with or without chemotherapy. Among these, 26.7% were positive to HPV. This group showed a better prognosis when compared to HPV-negative group. No genotyping is mentioned in this study. Similar results were achieved in 2023 by Ji (69) who focused on HPV-16 positive patients. In this study the HPV-positive patients accounted for the 16.7% of the 108 total patients included and 77.8% of these were HPV-16 positive.

The mentioned 2022 metanalysis by Shi (67) considers also the p16 status: p16 positive cancers cases showed an overall better prognosis. However, it is debated whether p16 should be considered a surrogate biomarker for HPV or an independent prognostic factor.

#### 4.9.3 Laryngeal cancer

Laryngeal cancer is the 20th cancer for incidence worldwide accounting for almost 200,000 new cases in 2022, and a 1.9/100,000 incidence (ASR), according to GLOBOCAN (8). It has a varied distribution globally, being more common in parts of Europe, Russia, Africa and Brazil.

Typical risk factors for laryngeal cancer include smoking, alcohol consumption, and exposure to industrial pollutants or asbestos. Chronic voice strain and frequent exposure to irritation from acids or chemicals can also increase risk.

Our database search retrieved 67 articles among which 13 reviews. Among the latter the more recent article is a metanalysis by Wang (70) published in 2020, including 11 studies and accounting for 1442 laryngeal squamous cell cancer patients. This analysis showed a trend of a better outcome for HPV-positive patients; however, it was not statistically significant. No HPV-positive percentage or genotyping was declared in the article. A previous metanalysis published in 2018 by Ahmadi (71) included 14 studies and accounted for 2,578 cases of laryngeal squamous cell cancer. They showed no difference in survival between HPV-positive and HPV-negative cases.

The most recent metanalysis focused on patients from India and was published in 2024 by Vani (72). The analysis included 34 studies on various head and neck cancers, including laryngeal cancer. In laryngeal cancer HPV-positivity was 29%. The lack of information on many confounders such as smoking or alcohol consumption prevented the Authors to achieve a clear conclusion on the causative role of HPV in laryngeal cancer.

Overexpression of p16 has been explored in a few studies included in the abovementioned metanalysis. However, it is highlighted that p16 has a low sensitivity and specificity when used as a surrogate biomarker for HPV (73, 74). In fact, p16 may have an independent prognostic role in laryngeal cancer (75, 76).

#### 4.9.4 Nasopharyngeal cancer

Nasopharyngeal cancer is the 23rd cancer for incidence worldwide accounting for approximately 120,000 new cases in 2022, and a 1.3/100,000 incidence (ASR), according to GLOBOCAN (8). It has a varied distribution globally, being more common in parts of Europe, Africa, China and South-Est Asia.

Risk factors for nasopharyngeal cancer include infection with Epstein-Barr virus, a family history of the disease, and certain ethnic backgrounds such as Southern Chinese or Southeast Asian descent. Lifestyle factors like smoking and alcohol consumption also increase the risk. Additionally, exposure to occupational hazards, such as dust and fumes, and a diet high in salted or preserved foods may contribute to the development of nasopharyngeal cancer.

Our database search retrieved 33 articles among which 3 reviews.

A 2024 metanalysis by Zhao (77) included 46 studies accounting for 6,314 nasopharyngeal squamous cell cancer patients. The major interest of this analysis was to determine prevalence of HPV in nasopharyngeal cancer patients. The global prevalence of HPV positive nasopharyngeal cancer was 0.18 (95% CI 0.14–0.23). When stratified by geographic region, prevalence was highest in North America (0.25, 95% CI 0.17–0.36), which is a non-endemic region for this malignancy. Asia, which an endemic area, had the lowest HPV prevalence estimate (0.13, 95% CI 0.08–0.22). HPV 16 (44%) and 18 (33%) were the predominant genotypes in found in nasopharyngeal cancer.

Although this metanalysis do not mention p16, other similar studies highlight that p16 may be correlated to prognosis in nasopharyngeal cancer but the association with HPV is elusive (78–80).

#### 4.9.5 Oral cavity cancer

Oral cavity cancer is the 16th cancer for incidence worldwide accounting for almost 400,000 new cases in 2022, and a 4.0/100,000 incidence (ASR), according to GLOBOCAN (8). It has a varied distribution globally, being more common in parts of Europe, Russia, India, Australia, USA and Brazil. Risk factors for oral cavity cancer include smoking, alcohol consumption, poor oral hygiene, chronic mouth infections, and a history of precancerous lesions also increase the risk. Additionally, age, male gender, and consumption of smoked or salted foods can contribute to the development of oral cavity cancer.

Our database search retrieved 22 articles among which 5 reviews.

The most recent systematic review on oral squamous cell cancer was focused on Europe. It was published in 2024 by Ghanem (81). However, in the article the HPV impact on the disease is not explored but just recalled from two other studies. In particular, from a 2023 metanalysis by Fonseca (82) and a 2022 metanalysis by Christianto (83). The former investigated the global prevalence of HPV in oral cavity squamous cell cancer. They found a significantly higher prevalence of HPV in the disease, with a pooled prevalence of 10%. The prevalence of HPV-positive oral cavity cancer varied geographically, with higher rates in North America, Northern Europe, and Oceania. The latter metanalysis investigated the impact of HPV infection on prognosis in oral squamous cell carcinoma. HPV positivity was associated with worse overall survival when compared to HPV-negative.

Previously, in 2022, Oliveira (84) published a metanalysis focused on South America. They included 38 studies accounting for 1643 squamous cell cancer patients. The pooled prevalence of HPV was 24.31% (95% CI 16.87–32.64). The pooled prevalence for HPV16 was 12.8% (95% CI 8.7–17.5). Thus, half of the HPV infections are form genotypes other than HPV-16. However, genotyping was not explored in this study. The highest pooled prevalence was observed in Venezuela (57.6%), and Chile had the lowest percentage (10.77%).

A worldwide systematic review and metanalysis was then published in 2023 by Katirachi (85). They included 31 studies comprising 5007 oral squamous cell cancer patients from 24 countries. The pooled prevalence of HPV positivity in oral cancer carcinoma was 6% (95% CI; 3%–10%). However, HPV-positivity varied greatly across countries with some countries such as Philippines, UK, South India, Korea, and France with 0% and others with higher prevalence, such as Jordan with a 37%. A study from India (86) was the only study that showed a statistically significant association of HPV infection with oral cavity cancer, with a prevalence of 13%.

The overexpression of p16 is sometimes considered across the many studies on oral cavity cancer. Overall, p16 expression does not appear to correlate with HPV and its role for prognosis varies (87, 88).

#### 4.9.6 Salivary glands cancer

Salivary glands cancer is the 28th cancer for incidence worldwide accounting for 55,000 new cases in 2022, and a 0.56/100,000 incidence (ASR), according to GLOBOCAN (8). It has a varied distribution globally, being more common in parts of Europe, Africa, North America, Australia and Brazil. Risk factors for salivary glands cancer include radiation exposure, previous head or neck cancer, and certain autoimmune conditions like Sjögren’s syndrome. Age and male gender also increase the risk. While the exact causes are unclear, genetic factors and exposure to industrial chemicals may play a role.

Our database search retrieved 4 articles among which no reviews.

The latest article issued on the topic was a case-control study (416 cases and 2080 matched controls) published in 2025 by Hung (89). They showed a significant difference in the prevalence of HPV infections between patients diagnosed with salivary glands cancer and the controls, with rates of 10.8% and 6.2%, respectively (p < 0.001), with OR = 1.885 (95% CI: of 1.315–2.701). The specific histological subtypes are not mentioned in this paper.

Previously a 2021 study by Mohamed (90) showed only 2 HPV-16 positive patients out of 16 patients with salivary glands cancer, with various histological subtypes, and the Authors claimed no association between HPV infection and the disease, based on such limited data.

A 2015 study published by Hühns (91) analyzed 200 patients with malignant and benign tumors of the salivary glands. The overall prevalence of HPV-infections was 10% and the distribution varied across different histological types. Most positive HPV derived from malignant tumors like adenoid cystic carcinoma and adenocarcinoma NOS and, to a lower proportion from acinus cell carcinoma, salivary duct carcinoma and adenoid basal-cell carcinoma.

A 2012 study published by Brunner (92) focused on p16 expression on 38 samples of minor salivary glands cancer. Only two cases resulted hrHPV positive and none lrHPV positive by ISH.

Only in the last-mentioned study p16 overexpression was considered. A p16 positivity of 71% (27/38) of the samples was found and these included the two HPV-positive cases, which showed particularly intense p16 positivity (92).

#### 4.9.7 Sinonasal cancer

Sinonasal cancer is a rare malignancy with incidence of 0.5/100,000 approximately.

Exact worldwide distribution and risk factors are not well characterized. Paradoxically, despite its aggressive histological features, sinonasal carcinoma tends to exhibit an indolent clinical course.

Our database search retrieved 50 articles among which 6 reviews.

The latest systematic review was published in 2025 by de Paiva (93) and included 32 articles, comprising 24 case reports, 4 cohort studies, and 4 case series studies, which reported a total of 101 cases.

Almost all cases (95.65%) were positive for hrHPVs. HPV 33 was the most prevalent type identified either by PCR (46.67%) or by ISH (47.83%). Other HPV genotypes detected were HPV-16, HPV-18, HPV-56, HPV-33, HPV-26, HPV-82. The Authors report a strong p16 positivity across the dataset for this kind of tumor.

#### 4.9.8 Tongue cancer

Tongue cancer is a cancer with estimated 20,000 new cases and a 3.6/100,000 incidence in the USA in 2025, according to SEER (8).

Risk factors for tongue cancer include smoking, alcohol consumption and betel nut use. Age, male gender, and a history of oral or head and neck cancers are additional risk factors.

Globally, the tongue is a typical localization of oral cavity cancers, accounting for approximately 25%–40% of cases at this site. Its incidence has been rising in both high and low income countries, particularly in the United States and parts of Europe, often in patients even without traditional risk factors (94).

Our database search retrieved 30 articles among which 4 reviews.

There is no systematic review for HPV in tongue cancer published in the literature. Among the 30 retrieved articles only a few analyze HPV prevalence or prognostic value specifically in tongue cancer. Many articles analyze tonsil cancer and base tongue cancer together; thus, the specific contribution of tongue cancer cannot be determined in these studies.

A recent study on 186 tongue squamous cell cancer patients from Thailand, published by Kritpracha (94) in 2025, showed an overall prevalence of HPV infection of 9.6%, with most common genotypes HPV-16 (14 cases, 7.5%), HPV-33 (3 cases, 1.6%), and HPV-18 (1 case, 0.5%). At the multivariate analysis HPV-positivity was correlated to a poorer prognosis when compared to HPV-negative cases.

A 2020 article by Alsofyani (95) studied 44 cases of tongue squamous cell cancer and identified HPV in 23% (10/44) of cases. Genotyping is not mentioned in the article although in the abstract it is claimed that the HPV positive cases are HPV-18. The survival analysis showed a worse prognosis for HPV-positive cases when compared to HPV-negative cases.

A 2018 article by Delgado Ramos (96) on 53 patients on a single institution in Ecuador showed an HPV prevalence of 41.5% (21/53 cases). Genotyping showed 18 different HPV types both low-risk and high risk. The most prevalent genotypes were the high-risk HPV-33 (4/21 cases; 19%) and the low-risk HPV-67 (4/21 cases; 19%). Other hrHPVs identified were HPV-58, HPV-35, HPV-56, HPV-52, HPV-45.

A 2017 article by Ashraf (97) analyzed 50 cases of tongue squamous cell cancer and showed an HPV prevalence of 14% (7/50 cases) of which none was HPV-16 or HPV-18 positive. No further genotyping was performed.

A 2013 study by Garcia-de-Marcos (98) considered 64 patients with tongue squamous cell cancer and found an HPV prevalence of 26.2% (16/61 cases, 3 cases excluded for insufficient DNA). All HPV-positive cases were high-risk genotypes but one, which remained undefined. Among the identified HPV-positive cases in 50% (8/16) was HPV-56, in 31.2% (5/16 cases) was HPV-18, in 12.5% (2/16 cases) was HPV-16, in 12.5% (2/16 cases) was HPV-66, in 6.2% (1/16 case) was HPV-39 and in 6.2% (1/16 case) was HPV-51.

In fact, a Japanese cohort of 32 young tongue squamous cell cancer patients showed 0% prevalence of HPV (99).

A 2011 case-control study by Elango (100) including 60 tongue squamous cell cancer cases and 46 normal oral mucosa controls detected HPV-16, with specific HPV-16 primers, in 48% (29/46 cases) of the cases and none of the controls. HPV positivity was associated with well-differentiated cancers (p = 0.041) and low recurrence rate (p = 0.014).

A 2011 survival analysis by Attner (101) on 87 tongue squamous cell cancer patients showed an HPV positivity prevalence of 78% (68/87 cases). Kaplan-Meier curves showed a better overall survival (p = 0.0004) and disease-free survival (p = 0.0008) for HPV-positive patients when compared to HPV-negative cases.

A 2010 study by Lee (102) on 36 patients diagnosed with oral tongue cancer and 25 normal controls showed HPV in 36% (13/36 cases) of cancer patients compared with 4% (1/25 cases) of the control. HPV-16 was the most common genotype, and its prevalence rate was 85% (11/13 cases). The remaining two cases were not genotyped. Of the HPV-16 infected oral tongue cancers, the integration rate of HPV-16 was 55% (6/11 cases).

Another 2010 study, by Attner (103), on 95 base tongue squamous cell cancer patients the HPV-positive tumors resulted 75% in prevalence, mainly HPV-16 (86%) followed by HPV-33 (10%).

In fact, prevalence was found as low as 1.96% (1/51 cases) in a 2008 study by Liang (104) on oral tongue cancer in a cohort of patients treated at the Mayo Clinic.

Finally, a 2004 study by Dahlgren (105) showed a 10.9% (12/110 cases) prevalence in mobile tongue cancer cases in Sweden. Among these, 9 resulted positive for HPV-16, 1 for HPV-33.

In the abovementioned Kritpracha (94) review p16 expression is considered as an unreliable surrogate marker for HPV (99). However, p16 overexpression appears to correlate to better survival rates (106).

#### 4.9.9 Tonsil cancer

Tonsil cancer is the most common oropharyngeal cancer, compromising 23.1% of all malignancies at this location, with incidence of 8.4/100,000. Risk factors for tonsil cancer include alcohol consumption, smoking, and a history of frequent throat infections. Age, male gender, and exposure to certain chemicals or pollutants can also increase risk.

Our database search retrieved 21 articles among which 2 reviews.

A 2025 metanalysis by Tao (107) included 102 studied on various head and neck cancers including tonsil cancer. The prevalence of HPV infection of any type for tonsil cancer was 28.14% (95% CI = 0.00%–64.53%, from 3 studies comprising 216 patients). The different histological subtypes of tonsil cancer are not declared in this metanalysis.

In fact, the studies on tonsil cancer focus mainly on prognostic factors and treatment. HPV positivity is recognized as a favorable prognostic factor. Genotyping was not usually performed.

This metanalysis do not consider p16 overexpression contribution to tonsil cancer. However, a recent review by Zupancic (108) highlights a possible role of p16 overexpression as a prognostic factor while remains inconsistent as a surrogate biomarker for HPV.

### 4.10 Hepatocellular cancer

Liver cancer is the 6th cancer for incidence worldwide accounting for approximately 860,000 new cases in 2022, and a 8.6/100,000 incidence (ASR), according to GLOBOCAN (8). It has a varied distribution globally, being more common in parts of Europe, Africa, China and South-East Asia. Risk factors for liver cancer include chronic hepatitis B or C infection, alcohol consumption, and cirrhosis of the liver. A diet high in aflatoxins and exposure to certain chemicals or toxins also contribute. Additionally, obesity, diabetes, and genetic conditions increase the risk of developing liver cancer.

Our database search retrieved a single article from 1992 and published by Scinicariello (109). Sixteen cases of hepatocellular carcinoma were analyzed and resulted in one positivity for HPV-16 and two for HPV-18.

No other article correlating HPV and hepatocellular cancer were found in the scientific literature.

The p16 status was not assessed in this study, although p16 overexpression has been correlated to poor survival in a systematic metanalysis by Lv (110).

### 4.11 Kidney cancer

Kidney cancer is the 14th cancer for incidence worldwide accounting for over 400,000 new cases in 2022, and a 4.4/100,000 incidence (ASR), according to GLOBOCAN (8). It is more common in high income countries. Risk factors for kidney cancer include smoking, obesity, high blood pressure, and a family history of the disease. Exposure to certain chemicals (e.g., cadmium and asbestos) may also increase risk.

Our database search retrieved only 2 reviews mentioning the correlation between HPV and kidney cancer.

A 2025 review by Zolfi (111) highlights previous research for the correlation of HPV with renal cell carcinomas. In 2014 Farhadi (112) analyzed 122 renal cell carcinoma specimens and detected HPV in 30.3% (37/122 cases) of them but only 4.1% (5/122 cases) in peritumoral normal tissue. Genotyping allowed the identification of HPV-16 in 5 (4.1%) cases, HPV-18 in 5 (4.1%) cases, and HPV-58 in 3 (2.4%) cases.

A 2012 study by Salehipoor (113) studied 49 patients with renal cell carcinoma and found a 14.3% (7/49 cases) hrHPV prevalence in cancer tissue while no HPV was found in control normal tissue. Genotyping showed 3 cases of HPV-16 and 4 cases of HPV-18.

A 1994 study by Kamel (114) detected a 52% (19/56 cases) prevalence in renal cell carcinomas. The most common genotype was HPV-33 (17 cases) followed by HPV-18 (16 cases).

In fact, a 2006 study by Hodges (115) on 62 renal cancers of various histology did not find any HPV in any of the samples.

A 2013 analysis by Khoury (116) using data from the Human Genome Atlas did not find any HPV positivity with RNA-Seq in renal cells carcinomas.

The aforementioned review by Farhadi (112) showed that p16 was detected in 24 (20.3%) cases. Data analysis showed a significant correlation between p16 expression and the presence of hrHPV (P < 0.001). It is unclear whether p16 expression can be correlated to prognosis in kidney cancer (117). Interestingly, the loss of p16 may have a role in carcinogenesis (118).

### 4.12 Lung cancer

Lung cancer is the 1st cancer for incidence worldwide accounting for almost 2,500,000 new cases in 2022, and a 23.6/100,000 incidence (ASR), according to GLOBOCAN (8). It has a varied distribution globally, being more common in parts of Europe, Russia and Asia. Risk factors for lung cancer include smoking, exposure to radon gas, and occupational inhalation of carcinogens like asbestos or chemicals. Secondhand smoke, a family history of lung cancer, and air pollution also increase the risk.

Our database search retrieved 95 articles among which 22 reviews.

The most recent systematic review was published in 2024 by Sequeira (119) including 97 studies comprising 9,098 patients worldwide with lung cancer of different histological subtypes including adenocarcinoma, squamous cell carcinoma, small-cell lung cancer, large-cell cancer, non-small-cell lung cancer. Varied HPV infection rates were detected in lung cancer, ranging from 0% to 69%. Numerous studies suggested an association between HPV and lung cancer, but the evidence remains inconclusive due to various limitations and differences across the studies analyzed, including the different histological subtypes.

Out of 97 studies only 24 performed at least a partial genotyping for HPV-16 and HPV-18, resulting in HPV-16 the most common genotype detected. Among these, only 3 studies performed and extended genotyping. Specifically, in a 2007 study by Park (120) on 112 lung cancer patients the prevalence of HPV-16, HPV-18, and HPV-33 were 10.7% (12/112 cases), 9.8% (11/112 cases), and 33.0% (37/112 cases), respectively.

In fact, a 2014 in a case control study by Anantharaman (121) comprising 3,083 lung cancer cases, of various histological subtypes, and 4,328 controls HPV was detected in 10% of the pooled tumor samples, 7% of which were positive for HPV-16, 1% to HPV-33, 0.6% to HPV-58, 0.3% to HPV-31, 1.1 to HPV-11, none expressed the viral oncogenes.

Similarly, a 2020 study by He (122) on 140 lung cancer patients, with various histologic subtypes, found a pooled HPV-positivity of 9.29% but the positive detection rate of HPV infection in the adjacent normal tissues was 11.43%. In the tumor tissue the specific genotypes of HPV in the 16 positive cases were: 43.7% (7/16 cases) HPV-16, 25% (4/16 cases) HPV-18, 18.7% (3/16 cases) HPV-42, 6.2% (1/16 cases) HPV-6, and 6.2% (1/16 cases) of multiple infection (HPV-18 and HPV-33).

Among the aforementioned studies, only the one by Anantharaman (121) explored p16 expression. The result was inconclusive for correlation to HPV presence. Specifically, 64 tumors were tested for p16 protein expression, 12 expressed low to medium levels (19%). Of these, 4 (1.4%) were also positive for HPV-16. Among the 52 tumors negative of p16 expression, 21 were HPV-positive (14 HPV-16-positive). Expression of p16 protein was also observed among 8 HPV-16 negative tumors (2.8%), reflecting background p16 expression.

### 4.13 Ovarian cancer

Ovarian cancer is the 18th cancer for incidence worldwide, the 7th in women, accounting for over 300,000 new cases in 2022, and a 6.7/100,000 incidence (ASR), according to GLOBOCAN (8). It has a varied distribution globally, being more common in parts of Europe, Canada, Russia and south-East Asia. Risk factors for ovarian cancer include a family history of the disease, inherited genetic mutations such as BRCA1 and BRCA2, and advanced age. Reproductive history factors, such as not having children or starting menopause late, also play a role.

Our database search retrieved 33 articles among which 7 reviews.

A comprehensive review on correlation between HPV and malignancies of the female upper reproductive tract was published in 2025 by Karachalios (123). This analysis included 12 studies on ovarian cancer of various histological subtypes, accounting for 996 patients. The pooled HPV prevalence was 15% (148/996 cases), ranging from 0% to 62%. HPV-16 was detected in 78.4% (116/148 cases) HPV-positive patients, HPV-18 in 17.5% (26/148 cases) and HPV-33 in 0.6% (1/148 cases). The correlation between histological subtypes and HPV positivity was not analyzed. No HPV was detected in the only study focused on fallopian tubes (124).

Similar results were found in previous metanalyses. For instance, the metanalysis published in 2021 by Cherif (125) on 29 studies comprising 2,280 ovarian cancer patients, with no histological subtypes declared, the prevalence ranged from 0% to 81% while the overall pooled prevalence was 16%. Across the studies a few genotypes were detected: HPV-16 (in 15 studies), HPV-18 (in 14 studies), HPV-33 (in 8 studies), HPV-45 (in 7 studies) in particular.

The overexpression of p16 was not explored in these studies. In one 2020 study by Yang (126) p16 appeared to be correlated to HPV and PD-L1 expression. In fact, p16 overexpression may have a prognostic role in ovarian cancer (127,128).

### 4.14 Prostate cancer

Prostate cancer is the 4^th^ cancer for incidence worldwide, the 2^nd^ in men, accounting for almost 1,500,000 new cases in 2022, and a 29.4/100,000 incidence (ASR), according to GLOBOCAN (8). It has a varied distribution globally, being more common in parts of Europe, Americas, Africa and Australia. Risk factors for prostate cancer include age, with higher risk in men over 50, family history of the disease, and African-American ethnicity.

Our database search retrieved 61 articles among which 15 reviews.

A 2023 metanalysis by Tsydenova (129) included 31 studies accounting for 1,607 tumor tissue samples and 1,515 control samples. HPV pooled prevalence in prostate cancer was 25.8%, while in normal tissue was 9.2%. The range of HPV prevalence in prostate cancer varied from 0% to 70%. The cumulative odds ratio of prostate cancer risk in HPV infection was 3.07 (95% CI:1.80–5.21). Various HPV genotypes were detected, including HPV-16, HPV-18, HPV-6, HPV-11, HPV-33, HPV-31 and others. Specific genotype prevalences were not stated.

A 2024 case-control study by Yin (130) showed that people who had HPV infections in the past had a odds ratio of 2.321 (95% CI: 2.097–2.568) of getting prostate cancer when compared to people who had never had HPV infections. This study was performed on 5,137 patients with prostate cancer and 15,411 controls. Among cases and matched controls, HPV infections were diagnosed in 743 (14.5%) and 1,069 (6.9%) patients, respectively.

None of the abovementioned papers specify the tumor histology. However, prostate cancer is almost exclusively an adenocarcinoma (131).

The overexpression of p16 was not explored in these studies. Nonetheless, p16 overexpression is often detected in prostate cancer and may have a role for prognosis (132).

### 4.15 Testicular cancer

Testicular cancer is the 27th cancer for incidence worldwide, the 3rd in men younger than 40 year old, accounting for approximately 70,000 new cases in 2022, and a 1.7/100,000 incidence (ASR), according to GLOBOCAN (8). It has a varied distribution globally, being more common in Europe, South-America and Australia. Risk factors for testicular cancer include a history of undescended testicles, family history of the disease, and certain genetic conditions. Age and being between 15 and 35 years old also increase the risk. Additionally, personal history of testicular cancer or abnormal testicle development can contribute to higher likelihood of developing the disease.

Our database search retrieved 7 articles among which 5 reviews.

A 2019 metanalysis by Garolla (133) included 25 studies on testicular infections due to various agents, accounting for a total of 265,057 patients with testicular germ cells cancer. Four of these studies were focused on HPV, accounting for 274 patients and 156 healthy controls. Pooling of results did not show any significant association between HPV and testicular cancer (OR = 2.79, 95% CI 0.84–9.29, p = 0.09). Non further research has been published on the topic since then.

This study did not explore the p16 status across the sample cases. The expression of p16 is rarely assessed in testicular cancer. However, its loss may be linked to testicular cancer carcinogenesis (134).

### 4.16 Thyroid cancer

Thyroid cancer is the 7th cancer for incidence worldwide, accounting for more than 800,000 new cases in 2022, and a 9.1/100,000 incidence (ASR), according to GLOBOCAN (8). It has a varied distribution globally, being more common in, North America, Australia, China, parts of Europe and South America. Risk factors for thyroid cancer include a family history of the disease and exposure to radiation. Iodine deficiency or excess and a history of thyroid nodules or goiter can also increase the likelihood of developing thyroid cancer.

Our database search retrieved 7 articles and no reviews.

The latest article was published in 2025 by Zhao (135) who studied the correlation between EBV and HPV infections as risk factors for thyroid cancer on 255 thyroid cancer patients. The HPV prevalence was only 0.7%, thus no correlation could be drawn.

A 2024 case report by Zhou (136) reported 3 cases of a rare histologic type, the squamous cells thyroid cancer. All 3 cases were positive for HPV, two cases for HPV-33 and one case for HPV-6.

A 2024 case-control study from Taiwan published by Yang (137) performed on 3,062 cases of thyroid cancer and 9,186 controls showed that there was a significant difference in the prevalence of any kind of prior HPV infections between patients with thyroid cancer and controls (15.3% vs. 7.6%, p < 0.001). The adjusted odds ratio of prior HPV infections for patients with thyroid was 2.199 (95% CI = 1.939–2.492).

A 2023 study by Kalavari (138) performed on 40 patients with thyroid cancer found a low prevalence of HPV (2.5%, 1/40 cases).

In fact, a 2022 study by Salim (139) on 36 patients with thyroid cancer, 40 thyroid adenoma patients, and 40 apparently normal thyroid tissues. The detection of HPV 16/18 in thyroid cancers was found in 72.2% of cancer cases, 35% in adenomas, and 27.5% of healthy thyroid tissues.

Similarly, a 2021 study published by Dialameh (140) on 82 thyroid cancer patients and 77 benign nodules revealed an HPV prevalence of 13.4% in the former and 3.8% in the latter, and the difference was statistically significant (p = 0.015).

No HPV was found in 30 nodules, with final various diagnosis including cancer, or adjacent tissues in a 2015 study published by Stamatiou (141).

The aforementioned study by Zhou (136) showed p16 positivity in all 3 presented cases, both squamous cell carcinoma and papillary carcinoma. The other studies do not explore p16 status. Thus, no speculation can be achieved on the role of p16 or its correlation with HPV in thyroid cancer.

## 5 Discussion

HPV is a well-known carcinogenetic agent (142) and exerts its deleterious activity on anogenital organs, particularly on the cervix and anus, where approximately 100% of cancers depend on the infection. Typically, HPV infects squamous cells and may lead to developing of premalignant lesions which can be classified as low-grade lesions (LSIL) of high-grade lesions (HSIL), based on the depth of involvement of the multiple-layered squamous epithelium. HPV may also infect and cause dysplasia in glandular cells. In this case, grading the premalignant lesion is more challenging because glandular epithelium is typically single-layered and the diagnosis of dysplasia must rely on morphologic or immunophenotypic characteristics, such as p16 staining (143). When invasive squamous cancer develops, it is graded into a three-tier system (well, moderate, poorly differentiated). Adenocarcinomas are more difficult to grade as multiple characteristics must be considered, such as HPV-status, p16 expression and pattern of proliferation and invasion (144). In recent years, a new grading system, the Silva system (145), has been proposed based on the pattern of invasion. This grading system may have better correlation to prognosis (146).

**FIGURE 2:**
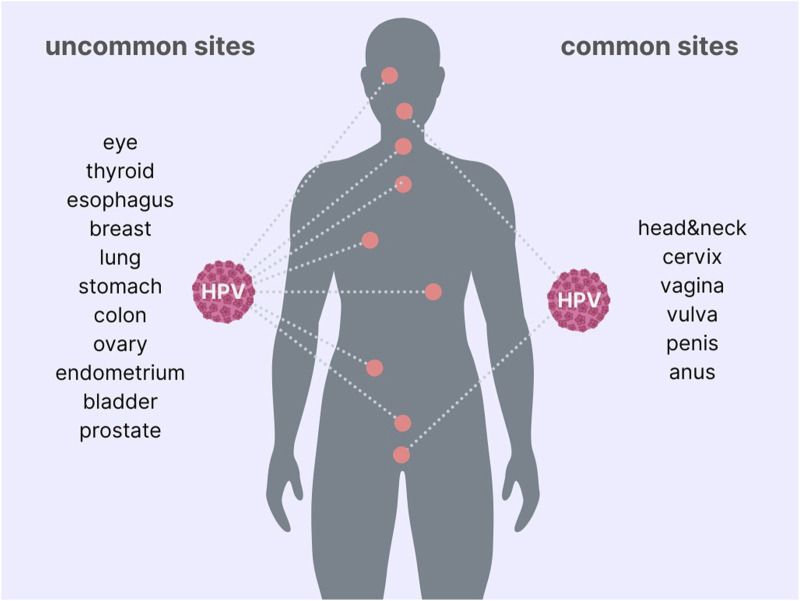
The figure shows common and uncommon sites of HPV-related cancers.

In fact, HPV may infect extragenital organs as well, particularly in the head and neck area (Figure 2). In many cancer types of the head and neck area, HPV is well-known prognostic factor (147). Moreover, HPV status, along with morphology and grading, are essential to differentiate tumor subtypes at the different anatomical sites. However, for reasons that have not been fully elucidated yet, some organs are more sensitive than others to the carcinogenic capacity of the virus. Because the virus spreads easily throughout the body also via self-inoculation it can reach several distant organs, although the route of infection is not always straightforward. At these levels, the infection may or may not be effective and productive, depending on the epithelia and the genotype-specific capacity. Thus, the sole detection of HPV-DNA in a specific type of cancer does not mean that the infection has a causative role in carcinogenesis for the specific organ. We generally rely on the prevalence of infection in a specific cancer type to hypothesize the causative role of HPV for that case. However, it cannot be excluded that the infection is just an associated event, and carcinogenesis is elicited by other molecular mechanisms. Thus, molecular evidence from *in vitro* and *in vivo* studies is advisable to verify the carcinogenic capabilities of HPV on specific tissues. Indeed, HPV E6 and E7 genes are successfully used to immortalize cells from various human organs, providing some insight that HPV may exert its actions on various human tissues (148–152). With these premises we reviewed the scientific literature trying to shed some light on the role of HPV carcinogenesis at uncommon locations for the infection.

Cancer sites for which we did not find any article were brain, parathyroid, hepatobiliary and pancreatic cancers. Thus, no conclusions can be reached on these organs, but it is unlikely that HPV would have a role in carcinogenesis at these sites. The reason why we did not find any publication for these sites can only be speculated. It is possible that it reflects a publication bias and negative results were not published while for other sites even unconclusive or negative results were indeed published.

For other organs, such as temporal bone and ear, eye, salivary glands, liver, kidney, testis and thyroid, the number of available articles is scant, and no conclusive result can be reached. Nonetheless, it is interesting that HPV can be found at such sites and in a few organs, such as ear and eye the HPV prevalence in cancer can be high, suggesting a causative role of the infection in carcinogenesis. Further studies are needed to clarify this association. However, these are rare diseases and it may be difficult to achieve a sufficient number of cases for statistically significant studies.

In a few cancers, such as larynx, tongue, tonsil, ovarian, and endometrial cancers, HPV has been often suggested as a causative factor although the percentage of HPV-positive cancer is generally small. The results we found are limited and inconclusive, and further research is required for these diseases. Interestingly, HPV seems to be more common in endometrial cancer with squamous differentiation (37). Moreover, a case report (136) on the rare squamous cells thyroid cancer showed 100% HPV-positivity in all 3 cases.

Many other cancers are unsuspected sites of HPV infection. In particular, lung, breast, prostate, stomach, and colon often showed varied and inconsistent results. Larger datasets are needed to clarify the role of HPV in these cancers, if any.

Correlations between HPV infection and breast cancer seemed elusive, although Rossi (21) stressed that there was a statistically significant difference in HPV prevalence between cases (24%) and controls (8%).

For colon cancer Damin (30) showed a significant OR of 4.054 (95% CI: 1.79–9.14) for HPV infection in the tumor samples compared with the nontumor controls, although 3 recent Iranian articles (31,32,153) found no correlation.

For gastric cancer the situation is similar, with a systematic review and metanalysis (59) that showed association between HPV infection and the risk of gastric cancer (OR = 1.53, 95% CI: 1.00–2.33) and recent results (61) that do not confirm the correlation.

Most convincing results were found with bladder cancer, although to a limited HPV prevalence of 19%, thanks to a few metanalysis published on the topic, including the one by Muresu (9) which reported an OR of 7.84.

Similar results for esophageal cancer, with an average prevalence of 24.8%, in which an increased risk for HPV infection, especially HPV-16 (OR = 2.69, 95% CI: 2.05–3.54 and OR = 2.35, 95% CI: 1.73–3.19) was shown (44).

An interesting and clear correlation between HPV and cancer is apparent for the sinonasal carcinoma (93).

Regarding specific genotypes, we must notice that only a few studies performed a full genotyping. Many studies are limited to the use of common HPV primers while others specifically investigate for HPV-16 and HPV-18. When genotyping is performed many other specific HPVs are found. HPV-16 and HPV-18 seemed to be the most common genotypes across all cancer sites. Besides sinonasal cancer, in which HPV-33 is typical, no conclusion can be made for other genotypes or cancer locations.

In this situation pancancer analyses and GWAS studies may help in better understanding the role of HPV in various cancers. For instance, a 2020 analysis, published by ICGC (154) with TCGA data from 2,658 samples across 38 cancer types, reported HPV-16 in cervical cancer and head-and-neck cancers, followed by HPV-18. HPV-33 was also identified in head-and-neck and cervical cancers. HPV-6 and HPV-45 were detected in bladder cancer.

In fact, a 2023 pancancer mendelian randomization study by Sun (155) reported HPV-16 as a risk factor implicated in the development of bladder cancer, colorectal cancer, and breast cancer, while HPV-18 was identified as a risk factor for prostate cancer, ovarian cancer, lung cancer and breast cancer.

Regardless the evidence of correlation between HPV infection and specific cancers, a vaccine strategy may protect all organs from the carcinogenetic capacity of the virus.

Recently, Cao (156) published a thorough umbrella review on HPV infection and the risk of cancer at specific sites other than anogenital tract and oropharyngeal region. Their search method allowed to include 31 studies involving 87 associations. The quality of the methodology was evaluated using the AMSTAR (157) method and the credibility of the evidence was assessed using GRADE (158). Their statistical analysis resulted into presenting the strength and credibility of the evidence in categories such as “convincing” and “highly suggestive”. The only association included into the “convincing” evidence category is the one between HPV-18 and breast cancer (OR: 3.48, 95% CI: 2.24–5.41), although it fell into the “very low credibility” category by GRADE. Eleven associations resulted as “highly suggestive”, these include oral squamous cell carcinoma, esophageal cancer, lung cancer, and breast cancer. All these associations fell into the “very low credibility” category by GRADE, except for breast cancer which was included into the “high credibility” category. Among the rest, 16, 41 and 18 associations were categorized as “suggestive,” “weak” and “non-significant” evidence, respectively. This work highlights the potential role of HPV carcinogenesis at anatomical sites other that the usual anogenital and oropharyngeal areas. It focuses on the data available to date, which is derived from a relatively small number of studies, often heterogenous and biased by weak evidence. This study compels the need for further research on this topic in order to build higher quality evidence and unquestionably define the actual role of HPV, if any, in many different cancer types throughout the body.

In our study we also explored the use of p16 overexpression as a surrogate biomarker for HPV positive cancers. In the retrieved dataset p16 has been often overlooked. Despite being considered as a useful biomarker in cervical cancer, for other cancer types there is inconsistent evidence that its overexpression is linked to HPV. However, p16 overexpression is detectable in 45% of human cancers while it is largely absent in normal tissue. Likely, p16 overexpression may rely also on different mechanisms than HPV carcinogenic action (159). For example, in head and neck cancers, p16 has been recognized as a prognostic factor even in HPV-negative cases (160).

The major limitation of our study is the heterogenicity of the data retrieved that derive from the multiple cancer sites, different histology, methods of analysis, and variable numerosity of samples. Thus, evidence might be weak or inconclusive for many cancer sites. However, given the well-known carcinogenic capabilities of HPV and the benefit that may derive from a broad vaccine strategy, further studies are advisable to better clarify the role of HPV infection at these uncommon cancer sites throughout the body where HPV has been detected.

## 6 Conclusion

HPV infection constitutes a risk factor for cancer developing at many sites throughout the body. Some cancer locations have been clearly correlated with HPV infections, for others the correlation remains elusive. Further research is required on the topic to better characterized such correlations. An anti-HPV vaccine strategy may be beneficial to protect all organs from the deleterious capacity of HPV of causing cancer.
